# Inferring mechanisms of response prioritization on social media under information overload

**DOI:** 10.1038/s41598-020-79897-5

**Published:** 2021-01-14

**Authors:** Chathika Gunaratne, William Rand, Ivan Garibay

**Affiliations:** 1 Department of Industrial Engineering and Management Systems, University of Central Florida, Orlando, FL 32816 USA; 2 Department of Business Management, North Carolina State University, Raleigh, NC 27695 USA

**Keywords:** Computational science, Information technology

## Abstract

Human decision-making is subject to the biological limits of cognition. The fluidity of information propagation over online social media often leads users to experience information overload. This in turn affects which information received by users are processed and gain a response to, imposing constraints on volumes of, and participation in, information cascades. In this study, we investigate properties contributing to the visibility of online social media notifications by highly active users experiencing information overload via cross-platform social influence. We analyze simulations of a coupled agent-based model of information overload and the multi-action cascade model of conversation with evolutionary model discovery. Evolutionary model discovery automates mechanistic inference on agent-based models by enabling random forest importance analysis on genetically programmed agent-based model rules. The mechanisms of information overload have shown to contribute to a multitude of global properties of online information cascades. We investigate nine characteristics of online messages that may contribute to the prioritization of messages for response. Our results indicate that recency had the largest contribution to message visibility, with individuals prioritizing more recent notifications. Global popularity of the conversation originator had the second highest contribution, and reduced message visibility. Messages that presented opportunity for novel user interaction, yet high reciprocity showed to have relatively moderate contribution to message visibility. Finally, insights from the evolutionary model discovery results helped inform response prioritization rules, which improved the robustness and accuracy of the model of information overload.

## Introduction

The rise of online social media as the dominant form of contemporary human communication has greatly changed the dynamics of information propagation. The heightened connectivity offered via social media platforms exposes users to high volumes and rates of information that they are not naturally equipped to handle. The inflow of information may reach an intensity beyond which the user’s typical cognitive abilities are hindered. This state is known as information overload^1^. Social media platforms attempt to overcome information overload by providing information scaffolds in the form of notification lists, news feeds, and summaries of what they believe should be the focus of a user’s attention. These information scaffolds emulate a sort of working memory^2–4^ of the user’s external-self^5^, by allowing information received to be stored, while perceived and responded to (or ignored). Eventually, individuals begin to rely on these information scaffolds provided by the social media platform to keep track and respond to their online counterparts.

However, despite the tools provided through the platform, users may still experience information overload. Importantly, information overload can characterize online conversations by constraining their volume and the number of participants engaged in discussion^6^. The responsiveness of overloaded social media users is generally less than the optimal responsiveness observed in their non-overloaded state. Analytical studies have supported this with evidence that for overloaded Twitter profiles, the decrease in responsiveness follows a power-law with the excess quantity of information received by the individual^7^ . Classical models of information diffusion, such as the independent cascade model^8–12^, linear threshold model^10,13,14^, and complex contagion model^15–17^, are unable to model the cognitive mechanisms of information overload, because information in these models are represented as a binary variable of awareness. Further, these models do not capture the properties that characterize social media messages and the users that create them. Yet, such properties amplify the prominence of certain received messages over others, and thereby their likelihood of being responded to^18^. Unlike in biological epidemics, only messages with a relatively high degree of prominence can produce a sustained chain of responses^19^. Recency^18–20^ and popularity of content^18^ have been shown to contribute to the prioritization of certain messages over others for response. However, identifying other factors contributing to response under information overload remains largely unexplored. In order to discover cognitively plausible mechanisms of information overload, models with rules encoding points within the space of possible interactions between these properties must be compared against one another. For instance, a more likely mechanism of response prioritization may involve both recency and poster’s popularity, rather than one or the other; i.e. a model where users respond to messages by the popularity of the posters of the most recently received messages, instead of one where messages are prioritized either by poster’s popularity or by how recently they were received.

In this study, we investigate several properties of social media messages that were hypothesized to have contributed to the visibility of messages received by overloaded users. In particular, we analyzed factors driving the response prioritization of highly active users of a cyber-vulnerability interest community engaged in cross-platform communication over GitHub, Reddit, and Twitter. As a result, we were able to identify properties that contributed to message visibility by users who managed to maintain high levels of activity, despite experiencing constant information overload. An existing information overload model (IOM)^6^ and the evolutionary model discovery framework^21^ were used for this purpose. In its basic form, the IOM stores messages in an actionable information queue, from which the oldest messages are removed under information overload. We modified the basic IOM by relaxing the assumption that under information overload, older messages are lost. Instead, the IOM was modified to assign each message a priority, according to a utility function. We implemented IOM within an agent-based model of conversation, the multi-action cascade model (MACM)^22^. The MACM simulates the flow of information within a network of agents acting on conditional probabilities of social influence extracted from data using marginal transfer entropy^23^. MACM is able to simulate cross-platform social influence by abstracting platform events into conversation-related actions as motivated by conversation theory^24^. The MACM+IOM coupled model was then used to simulate a month’s activity of the eight most active Twitter users in a cyber-vulnerability interest community, under the cross-platform, social influence of users from GitHub, Reddit, and Twitter.

Using evolutionary model discovery a framework for automated causal inference in agent-based models^21,21,25^, we tested nine factors which we hypothesized to affect the message prioritization process: conversation popularity, conversation size, initiator’s popularity, shared interests, reciprocity, URL popularity, URL familiarity, information expertise, and recency. Evolutionary model discovery performs random forest feature importance on genetically programmed agent-based model rules, to identify important causal factors and their optimal presence within the rule. Utility functions of the hypothesized factors were automatically evolved with evolutionary model discovery, exploring the vast space of possible rules for message prioritization, and the resulting data was analyzed by the random forest for factor importance.

Our findings show that messages received (1) more recently, (2) on conversations initiated by less globally popular users, (3) presenting a novel interaction with historically less interacted user, and (4) yet presented a higher chance of reciprocity were more likely to be prioritized for response under information overload. These simulation findings explain phenomena observed through analytical studies in the literature, in particular, the social factors determining message visibility for information epidemics^18,19^, the importance of recency in global information cascades^19,20^, and the observation that information popular on a global scale tends to be locally unpopular^26,27^.

## Methodology

A data-driven, agent-based model was used to investigate message visibility as their priority for response assigned by social media users experiencing information overload. In particular, agents of the MACM were made to use the IOM to determine which messages were to be prioritized over others for response, when experiencing information overload. The mechanism governing the response prioritization was then explored through evolutionary model discovery, a causal inference framework for agent-based models^25,28,29^. Through evolutionary model discovery, we evolve prioritization mechanisms towards those that produce simulations with the closest overall responsiveness to that observed in social media data, and analyze the importance of factors hypothesized to affect the decision to prioritize certain messages over others.

### Data

Data on the activity of users engaged in discussions and development related to cyber-vulnerabilities on GitHub, Reddit, and Twitter between the 1st of February, 2017 to the 1st of May, 2017 were considered for this experiment. Discussions were collected by querying user events on each platform that mention known CVE numbers (https://cve.mitre.org/cve/) along with events that contribute and share such events. The data was split into a calibration and a discovery period, from 1st of February, 2017 to the 1st of April, 2017 (458,098 unique events), and from 1st of April, 2017 to the 1st of May, 2017 (429,378 unique events), respectively. Each event in the dataset consisted of the following information: (1) time of event, (2) anonymized user identifier, (3) the anonymized event identifier of the immediate parent event to which this event responds to if any, (4) keywords identifying the information being discussed by the individual, and (5) domains of any external URLs referred to in the event.

### Model description

We studied response prioritization through the investigation of agents embodying the information overload model (IOM)^6^ and interacting with one-another according to the multi-action cascade model (MACM) of conversation^22^.

#### Modeling information overload

In the IOM, messages encoding information regarding the online activity of an individual’s influencers are stored in an *actionable information queue*, *A*. Messages are stored in *A* in the order with which they were received; i.e. older messages towards the end and newer messages towards the beginning. The current capacity of *A*, at time *t*, represents an individual’s attention span, $M_t$, as defined in^30^. Each message *m* has probability $p_{m}$, described later, with which it may be acted upon and removed from *A* at a given timestep. If an agent receives more messages than it can process, the actionable information queue can exceed its maximum capacity $M_{max}$. At this point, the user is considered to experience information overload and $M_{t}$ is reduced by a number of units that follows a power-law with the magnitude of information overload experienced, as observed in other analytical studies^7^. The new attention span, is calculated as shown in Eqs. (1) and (2).

1 $$\begin{aligned} M_{t}= & {} \left\{ \begin{array}{@{}ll@{}} M_{t-1} - O_{t-1}^\alpha , &{} \text {if}\ O_{t-1}^\alpha <= M_{t-1} \\ 0, &{} \text {otherwise} \end{array}\right. \end{aligned}$$

2 $$\begin{aligned} O_{t-1}= & {} \left\{ \begin{array}{@{}ll@{}} (|A_{t-1}| + |R_{t-1}|) - M_{max}, &{} \text {if}\ |A_{t-1}| + |R_{t-1}| >= M_{max} \\ 0, &{} \text {otherwise} \end{array}\right. \end{aligned}$$

where *R* is the list of messages received this timestep and $\alpha$ is the rate of attention loss, a parameter of the model. Since $M_{t} < M_{t-1}$ when a user is overloaded, and the older messages are stored toward the end of *A*, older messages are truncated when the actionable information queue is reduced to length $M_{t}$. In other words, IOM in it’s basic form prioritizes on recency, i.e. message visibility is determined by the chronological order in which messages were received, with the oldest, not-responded-to messages having a higher likelihood of never gaining a response as an agent experiences information overload.

#### Multi-action cascade model

The multi-action cascade model is an information theoretic agent-based model of conversation^6,22^. According to Pask^24^, information is exchanged between individuals through the act of conversation. Individuals engaged in conversation continually query one-another in order to further their own understanding of a particular topic, or express their own understandings/opinions. Unfortunately, most diffusion of information models in the literature instead simulate the spread of information as the diffusion of a binary awareness regarding a topic. This makes it difficult to model actions taken by individuals that seek to respond to received information; for example, replies in Twitter.

Instead, the MACM redefines the representation of information passed in conversations, allowing for the ‘growth’ of tree-like conversations by performing more than simple act of sharing. Conversations are structured as trees, with each conversation-node representing an event performed by a user through which information was added to the conversation. Agents can perform (1) initiation events, the creation of a root conversation-node; (2) contribution events, the addition of a conversation-node with new content; or (3) sharing events, the addition of a node with content copied from an existing node from the current conversation-tree or another conversation-tree. Accordingly, as the agents perform these events, messages that represent social media notifications are passed through the network to the neighbors. These messages are represented by a tuple of four elements, (1) the sender (also referred to as the influencer), (2) the action taken, (3) the node in the conversation to which the action has been taken, and (4) the content of the message conveyed by the individual.

A relationship can then be defined between two individuals by describing how likely a message of a particular action type is to influence another user to generate a message of a particular action type. Thus, the three action types allow for an influencer $\rightarrow$ influenced edge, say *V* influencing *U*, to have nine possible relationships of social influence in one direction: ($V_{initation} \rightarrow U_{initiation}$, $V_{initation} \rightarrow U_{contribution}$, $V_{initation} \rightarrow U_{sharing}$, ...,$V_{sharing} \rightarrow U_{sharing}$). The MACM, extracts a directed network of influence probabilities from the activity data, by calculating the ratio between the marginal transfer entropy, from the event timeseries of *V* performing event *x* to the timeseries of *y* events by *U*, to the marginal entropy of the event timeseries of *U* performing event *y*, when $V_{x}=1$ and $U_{y}=1$, as defined in Eq. (3) (1: event occurred, 0: no event occurred). In the model, when an agent *U* receives a message that *V* performed *x*, the corresponding conditional probability, $P(U_y|V_x)$, is then referred to decide whether to respond to the message with event *y*.

3 $$\begin{aligned} P(U_{y}|V_{x}) = \frac{TE_{V_{x}=1 U_{y}=1}(V_{x} \rightarrow U_{y})}{H_{U_{v}=1}(U_{y})} \end{aligned}$$

#### Model parameters

**Figure 1:**
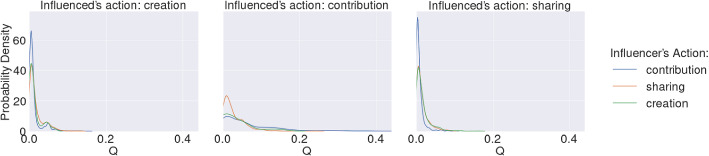
Probability density functions of conditional probabilities $P(U_y|V_x))$. Probability densities of influenced individual *U* performing action *y* given influencer *V* performs action *x* over users in the calibration data. The long-tailed distributions illustrate the heterogeneity of $P(U_y|V_x))$ among all existing relationships, with a few relationships having high conditional probabilities of action and many having relatively lower probabilities of action. This heterogeneity was highest for social influence on the influenced individual’s contribution actions, i.e. the probability density functions for $P(U_y|V_x))$, where $y=contribution$, had a much longer tails.

As shown in Fig. 1, the distributions for $P(U_y|V_x))$ on the calibration dataset were long-tailed, i.e. while many relationships demonstrated lower conditional probabilities, some strongly influential relationships existed. Additionally, the degree of social influence on contribution actions follows a longer tail and is more widely distributed than for initiation or sharing actions. This meant that it was inappropriate to represent this heterogeneity by calculating a central, representative conditional probability for all relationships. Instead, we calculated the conditional probabilities for each relationship in the MACM network, per $V_x\rightarrow U_y$ pair. Additionally, the IOM has two parameters $\alpha$ and $M_{max}$ for which 0.8 and 30 were used, respectively, as these values had been previously identified as optimal^6^.

### Evolutionary model discovery of response prioritization

We perform evolutionary model discovery^25,28^ of the MACM+IOM coupling described above to analyze the importance and optimal presence of a set of factors hypothesized to drive the response prioritization by overloaded social media users. Evolutionary model discovery has been successfully applied to the Artificial Anasazi model^31,32^ to uncover factors important to the socio-agricultural decision-making of an ancient Pueblo civilization. The framework functions in two stages: (1) evolving the best combination of hypothesized factors and operators to a rule of interest in a given agent-based model with with genetic programming and (2) training a random forest on the factor presence to fitness data produced by the genetic program and analyzing factor importance with feature importance estimation.

Genetic programming has been used in the past to develop agent rules^29,33–35^. However, when trying to extract the human decision-making processes that produce complex macro-scale patterns in data, a global optimum is not always easily reachable or apparent. Instead, through the statistical analysis of the data produced by the optimization process, inferences can be made to identify the important causal factors; in particular, the factors that the model is most sensitive to and their optimal presence in the agent-rule. Through random forest importance analysis on the factor-presence to fitness data, the factors most important to the prediction of the model fitness, can be identified, Statistical tests on the presence of the most important factors can then be performed to identify their optimal presence values. Finally, these insights can be used to construct more robust and accurate agent rules.

#### Message prioritization

By sorting messages in the order in which they were received and through the truncation of older messages upon information overload, the original model essentially prioritizes the most recent messages, $F_{Recn}$. We generalize this process of message prioritization with a utility function, *u*(*m*). Accordingly, the IOM originally uses the prioritization utility given in Eq. (4).

4 $$\begin{aligned} u(m)=F_{Recn}(m) \end{aligned}$$

Under information overload, messages on the actionable information queue and newly received messages would be combined and sorted according to their *u*(*m*) scores, and messages would be dropped in increasing order of *u*(*m*), until $M_t$ messages were left to form the new actionable information queue.

#### Hypothesized factors

Nine factors were hypothesized to drive the response prioritization of highly active users under information overload:

1. Conversation popularity $F_{ConvPop}$: Conversation popularity represents the global popularity of a particular information cascade. It is measured by the normalized number of users that have responded to the conversation created by the root message that was created by the original poster.
2. Conversation size $F_{ConvSize}$: Conversation size represents the global volume of a particular information cascade. It is measured by the normalized number of responses that have accumulated to the root message that was created by the original poster.
3. Initiators popularity $F_{InitPop}$: Initiator’s popularity represents the global popularity of the conversation initiator. This is measured as the number of times messages by this individual had been responded too by any individual.
4. Common conversation interactions $F_{Intr}$: Common interactions measures the number of times the individual has participated in a conversation which the other user has also participated in.
5. Absolute reciprocity $F_{Recip}$: Absolute reciprocity (both positive and negative) was measured as the number of times the individual has responded directly to an another individual.
6. URL domain popularity $F_{URLPop}$: URL domain popularity represents the global popularity of any URL domains that were mentioned by all users. It is measured as the normalized count of messages that have a reference to the URL domain.
7. URL domain familiarity $F_{URLFam}$: URL domain familiarity represents the local popularity of any URL domains, measured as the number of references this individual has made to the URL domain in their past messages.
8. Information expertise $F_{Info}$: Information expertise represents how often this user mentions a particular piece of information.
9. Recency $F_{Recn}$: Recency was measured as the reciprocal of the amount of time that had passed since the message was originally received by the individual.

These factors were implemented as functions in the agent-based model and used as primitives of the genetic program. Each function returned a normalized sub-utility score in the range (0, 1]. The genetic program evolved a syntax tree representation, with the factors as terminal nodes, connected by operator nodes. Recent work in behavioral economics^36^ has shown that emotional and social dimensions play a role in decision-making. Accordingly, while responding based on higher $F_{Recn}$ alone may be explained by the notification ordering imposed by the social media platform, the rest of the factors above allow for the construction of behaviors that are explained via social theories. In particular, the factors listed above can be related to different theories. A high emphasis on $F_{ConvPop}$, $F_{ConvSize}$, $F_{InitPop}$, or $F_{URLPop}$ would demonstrate observational learning as explained in social learning theory^37–39^ and also agree with status theory^40,41^. A high emphasis on $F_{Recip}$ is related to social exchange theory^42^. Emphasis on higher $F_{Info}$ would indicate novelty-aversion, a low $F_{Info}$ would indicate novelty-seeking behavior. Finally, high $F_{Intr}$ would display homophily, agreeing with social correlation theory^43,44^, while low $F_{Intr}$ would indicate heterophilous behavior. However, in our model discovery process we do not constrain rule formation to those that conform to established social theories, and instead allow for the evolutionary aglorithm to discover rules that best fit the data.

#### Operators

Operators were used in the genetic program to combine the factor primitives to form utility functions, included addition ($+$), subtraction (−), multiplication ($\times$), and division ($\div$). Inclusion of $\times$ and $\div$ allowed for the creation of more complicated factor interactions, each which had to be treated as separate factors when performing the factor importance analysis.

#### Experimental setup

16 of the most active users in the dataset described above with the highest number of retweets were chosen as the most active Twitter users. Eight of these users were used for evolutionary model discovery (training users), while the other eight were kept for validation of the evolutionary model discovery results (holdout users). As described above, the conditional probabilities of influence to and from these 16 users and the data necessary for the 9 factors above were calculated on the calibration dataset, using a Python CUDA-GPU implementation of the marginal transfer entropy to marginal entropy ratio calculation in Eq. (3). A mean of 260.625 (std dev. 98.217) non-zero relationships from influencing user profiles were discovered for the 8 training users in the calibration period.

The MACM+IOM model was implemented in NetLogo^45^. For each simulation in evolutionary model discovery the training users were simulated, inputting their conditional probability data. The simulations were run for the discovery period, 1st of February, 2017 to the 1st of April, 2017. For each tick (1 tick represents 1 h), the events performed by the influencers of the simulated agents were injected into the agents as received information and processed as described above (“Modeling information overload”). Data required for the evaluation of the factors described above were calculated beforehand on the simulation period data and input to the NetLogo simulations (see Supplementary Information for summary statistics). Events were removed from the actionable information queue either due to overload, as explained above, or due to successful propagation after response, accordingly.

The fitness of each simulation to the data was calculated as the accuracy of the overall user responsiveness simulated. This was calculated as follows. Messages received by each simulated user, *U*, during the simulation period were extracted beforehand from the activity data of users influencing the simulated users during simulation period. The actual number of responses to each of these messages, *m*, were counted from the activity data of each *U* during the simulation period, $R^{real}_{U_m}$. When the simulation was run, each *m*, was input into the simulation as received information to the corresponding simulated user, *U*, at its respective simulation tick. The number of simulated responses to each *m* per *U* was recorded, $R^{sim}_{U_m}$. The root mean squared error (RMSE) between the simulated and actual responses to influencer messages was then calculated as shown in Eq. (5).

5 $$\begin{aligned} RMSE = \sqrt{ \frac{1}{|m_{all}|}\sum _{m \in m_{all}}{(R^{real}_{U_m} - R^{sim}_{U_m})^2}} \end{aligned}$$

Thirty genetic program runs were conducted to evolve the message prioritization utility function with the factors and operators described above. The RMSE of responsiveness was used as the fitness function. Crossover rate was set to 0.8, mutation rate was set to 0.1, and a population size of 50 was used. Each run was evolved for 50 generations. A tree representation was used, with the terminal nodes being the hypothesized factors, combined via primitives that represented the operators listed above. Rules were derived by simplifying the resulting trees, and the *presence* score of a factor was considered as its coefficient in the simplified rule. In order to avoid *bloat*^46^, a maximum depth of 10 was set for the evolved factor trees (see Supplementary Information for an example). As all operators used were binary, a maximum of $2^{10}$ terminals were possible in a rule, allowing for rules with all 9 factors, and maximum presence score range of $[-1022,1024]$. The individuals of the initial population were generated with randomly using the ramped half-and-half algorithm. The best values for $\alpha$ and $M_{max}$ for MACM+IOM on the described dataset, 0.8 and 80, were used, respectively, after calibrating the two parameters to the data^6,7^. The random forest was trained on the data produced by the genetic program with the CART (Classification and Regression Trees) algorithm. Gini and permutation accuracy importance^47^ were used to find the importance of the factor and factor interactions selected by the genetic program towards the prediction of the fitness of the simulations during the evolutionary search. Mann–Whitney U tests were used to test for significant difference in the permutation accuracy importance. Next, the presence of the most important factors were analyzed.

Finally, the results were validated by testing several models with and without the important factors at their optimal presence, as identified by evolutionary model discovery. The eight holdout users were used for this purpose as a check for robustness of the results. RMSE of 100 runs of each model executed over the simulation training period were compared with one another.

## Results

**Figure 2:**
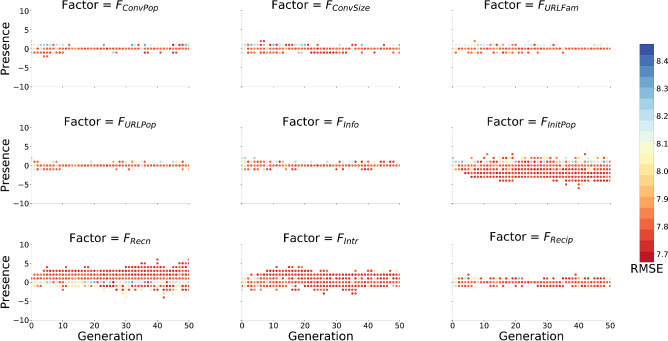
Selection of primitives by generation with the simulation RMSE produced. Causal factors that had higher contribution to response prioritization are expected to have higher selection pressure on the genetic program, exhibiting increasing negative or positive bias in presence within *u*(*m*) over generations. Results have been averaged over the 20 genetic program runs and red indicates lower RMSE. $F_{InitPop}$, $F_{Recn}$, and $F_{Intr}$ show a gradual increase the different presence values in *u*(*m*) explored by the genetic program in comparison to other factors considered.

Some example rules, along with their RMSE, produced by the genetic program are shown in Table 1, where the 20 rules that produced the lowest RMSE are provided. Most of the highest fit rules appeared to contain $F_{Recn}$. Figure 2 shows the selection of factor primitives by the genetic program as the generations progressed, along with the RMSE of the respective simulations. Certain factors showed to have higher selection pressure than others; $F_{Recn}$ had a relatively stronger positive selection, along with a weaker positive selection for $F_{Intr}$. $F_{InitPop}$ showed a relatively stronger tendency to be selected with negative presence.

**Table 1:** Examples of rules that produced the lowest RMSE in responsiveness to the real-world data.

Rule	RMSE
$u(m)=-F_{InitPop}(m)+F_{Recn}(m)$	7.6597
$u(m)=-F_{InitPop}(m)$	7.6669
$u(m)=-4F_{InitPop}(m)+3F_{Recn}(m)$	7.6674
$u(m)=F_{Recn}(m)$	7.6706
$u(m)=F_{Recn}(m)-1F_{Intr}(m)$	7.6750
$u(m)=-2F_{InitPop}(m)+F_{Recn}(m)+2F_{Recip}(m)$	7.6751
$u(m)=F_{ConvPop}(m)+2F_{Recn}(m)$	7.6751
$u(m)=2F_{Info}(m)-4F_{InitPop}(m)+2F_{Recn}(m)$	7.6758
$u(m)=-2F_{InitPop}(m)$	7.6761
$u(m)=F_{Recn}(m)$	7.6765
$u(m)=-F_{InitPop}(m)+F_{Recn}(m)$	7.6768
$u(m)=-2F_{InitPop}(m)+3F_{Recn}(m)$	7.6769
$u(m)=[F_{InitPop}(m), F_{Recn}(m)]$	7.6776
$u(m)=-F_{InitPop}(m)+F_{Recn}(m)$	7.6783
$u(m)=-2F_{InitPop}(m)+F_{Recn}(m)+F_{Intr}(m)$	7.6783
$u(m)=[F_{InitPop}(m), F_{Recn}(m)]$	7.6786
$u(m)=-F_{InitPop}(m)+F_{Recn}(m)$	7.6787
$u(m)=-F_{InitPop}(m)+F_{Recn}(m)$	7.6792
$u(m)=-2F_{InitPop}(m)-1F_{Intr}(m)$	7.6798
$u(m)=-4F_{InitPop}(m)+2F_{Recip}(m)$	7.6805

The top 20 rules that resulted in the lowest RMSE are provided as examples of rules produced by the genetic program in evolutionary model discovery. Solutions for *u*(*m*) were evolved by combining the nine factors using the $+$, −, $\times$, and $\div$ operators. The presence value of a factor $F_i$ is considered as its coefficient in *u*(*m*). $F_{Recn}$ and $F_{InitPop}$ appear in most of the optimal solutions. (Square brackets indicate interactions between factors i.e. multiplication or division).

**Figure 3:**
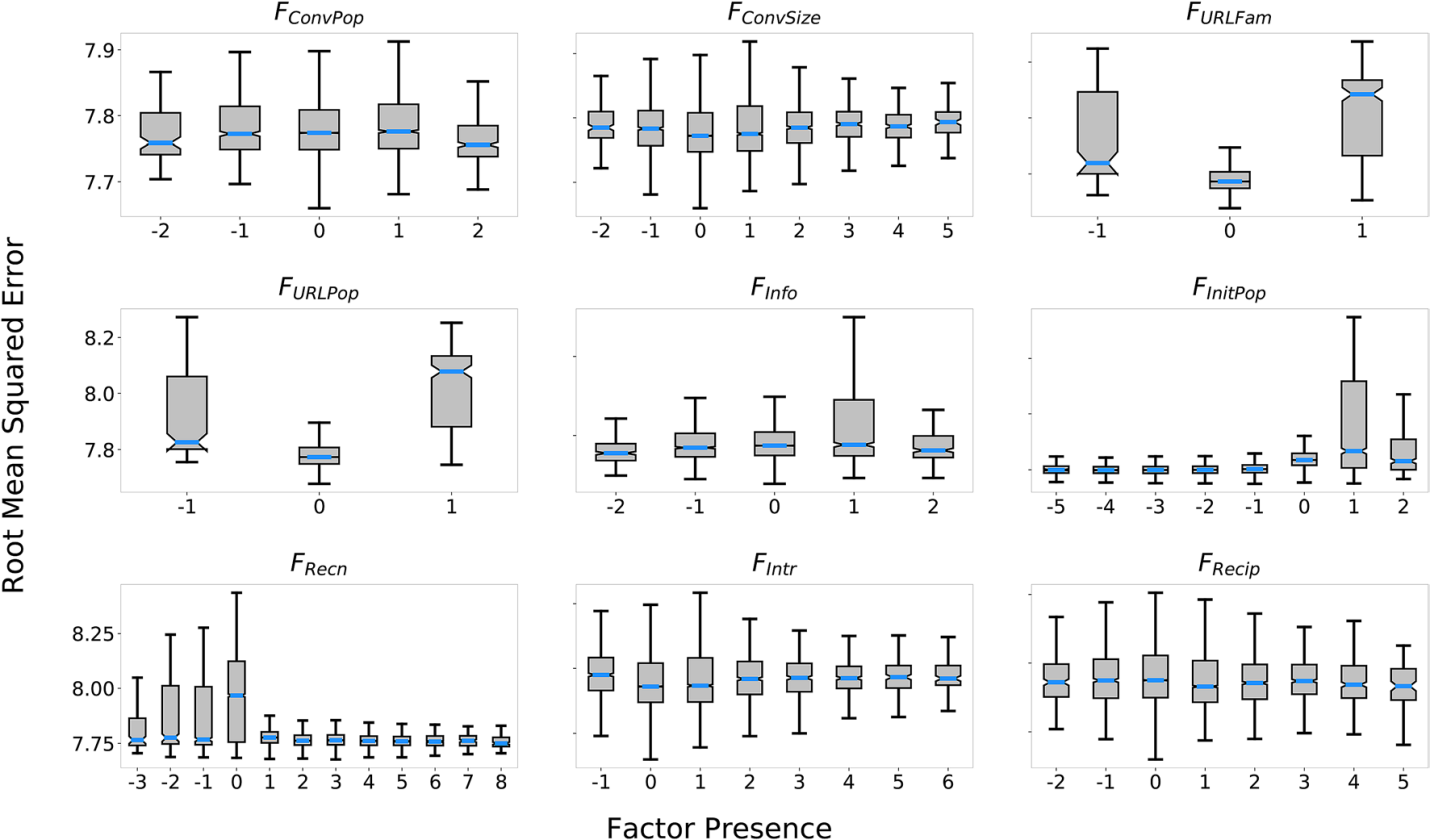
Marginal RMSE of responsiveness by factor presence of simulations of the models generated through the genetic program. $F_{Recn}$ shows significantly lower RMSE when positively present in *u*(*m*) and $F_{InitPop}$ shows significantly lower RMSE when negatively present in *u*(*m*). Both content URL related factors, $F_{URLPop}$ and $F_{URLFam}$ show significantly lower RMSE when absent from *u*(*m*). In order to reduce statistical bias, only factor presence values that appeared at least 100 times in the genetic program individuals are displayed.

Figure 3 displays the marginal RMSE of responsiveness under different presence values for each factor. Only presence values that appeared at least 100 times in the rules generated through the genetic program were considered here, as well for the rest of the analysis, to account for the variance caused by the stochasticity of the agent-based simulations and ensure a reasonable estimate of the corresponding RMSE. From observation, it is apparent that RMSE is lowest when presence of $F_{URLFam}$ and $F_{URLPop}$ is 0, i.e. when absent from the model. RMSE is lowest when $F_{InitPop}$ is present negatively and $F_{Recn}$ is present positively.

**Figure 4:**
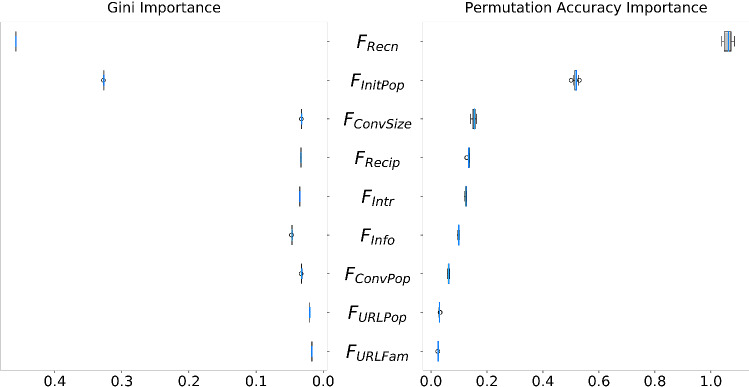
Gini and Permutation Accuracy importance of factors hypothesized to drive response prioritization under information overload. $F_{Recn}$ is by far the most important factor for response prioritization, more than twice as more important than the second most important factor, $F_{InitPop}$. The rest of the factors are ordered in descending order of importance as follows: $F_{Info}$, $F_{Intr}$, $F_{Recip}$, $F_{ConvPop}$, $F_{URLPop}$, $F_{ConvSize}$, $F_{URLFam}$.

**Figure 5:**
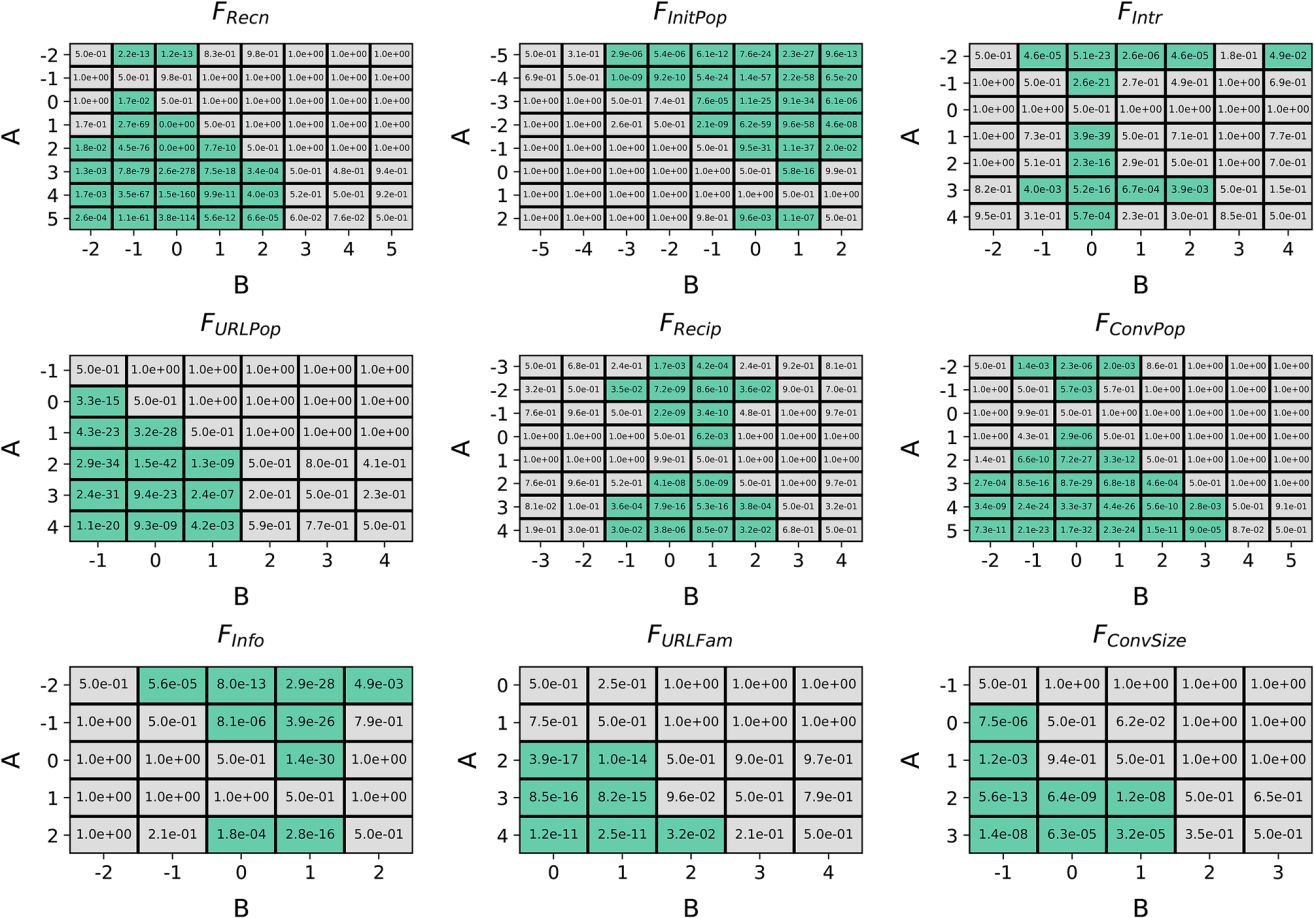
Optimal presence scores for hypothesized causal factors. P values of systematic one-tailed Mann–Whitney U tests between presence values of the nine hypothesized factors for the alternate hypothesis: $\text {RMSE for presence }A < \text {RMSE for presence }B$ (null hypothesis: $\text {RMSE for presence }A = \text {RMSE for presence }B$) for $\text {original significance level}=0.05$ with Bonferroni corrections applied for all comparisons within each factor. Green cells indicate agreement of the alternate hypothesis. $F_{Recn}$, $F_{ConvPop}$, $F_{ConvSize}$, $F_{URLPop}$, $F_{URLFam}$, and $F_{Recip}$ are optimal at positive presence within *u*(*m*), while $F_{InitPop}$ and $F_{Info}$ are optimal when present negatively within *u*(*m*). $F_{Intr}$ is optimal at both negative and positive presence values.

Figure 4 displays the Gini and permutation importance of nine hypothesized factors towards the random forest’s ability to re-predict the RMSE of the discovery data produced by the genetic program. Both techniques agreed on the relative ordering of importance of the hypothesized factors. $F_{Recn}$ shows to have the highest importance by far, more than twice as high as the second most important factor $F_{InitPop}$. Systematic one-tailed Mann–Whitney U tests (Fig. 5) of the alternate hypothesis that permutation importance of *A* > permutation importance of *B* (null hypothesis: permutation importance of *A* = permutation importance of *B*) at $\text {original significance level}=0.05$, show that there is a clear statistically significant ordering of the factors according to permutation importance; in descending order of permutation (and gini) importance: $F_{Recn}$, $F_{InitPop}$, $F_{Info}$, $F_{Intr}$, $F_{Recip}$, $F_{ConvPop}$, $F_{URLPop}$, $F_{ConvSize}$, $F_{URLFam}$.

Figure 5 displays the results of systematic one-tailed Mann–Whitney U tests for each factor testing the null hypothesis: $\text {RMSE for presence }A < \text {RMSE for presence }B$ for $\text {original significance level}=0.05$ (with Bonferroni corrections applied to all comparisons within each factor). Green cells indicate where each Mann–Whitney U test passed. The optimal presence values within *u*(*m*) for each factor observed through this comparison are as follows: $F_{Recn}$: + 3, + 4, + 5; $F_{InitPop}$: − 4, − 5; $F_{ConvPop}$: + 4, + 5; $F_{ConvSize}$: + 2, + 3; $F_{URLPop}$: + 2, + 3, + 4; $F_{URLFam}$: + 4; $F_{Info}$: − 2, $F_{Recip}$: − 2, + 3, + 4; and $F_{Intr}$: − 2. In other words, recency, conversation popularity, conversation size, URL popularity, URL familiarity, and reciprocity had positive effects on message prioritization, while the popularity of conversation initiators and information expertise had negative effects on message prioritization. Common conversation interactions had equal positive and negative effects on message prioritization. The magnitude of the optimal presence values are seen to have some positive correlation with the factor’s importance, which was expected as the genetic program would have selected more strongly for factors with higher contribution to fitness. Together with the factor importance results, this indicates that, in decreasing order of importance, message prioritization by overloaded users was most likely driven by preference for: (1) more recent messages, (2) conversations initiated by less popular users, (3) novel content, (4) either senders with higher similarity or higher difference (over those with moderate similarity/difference), (5) senders that have shown the receiver higher absolute reciprocity, (6) more popular conversations, (7) more popular URLs in content, (8) larger conversations, and, least importantly, (9) more familiar URLs in content.

**Figure 6:**
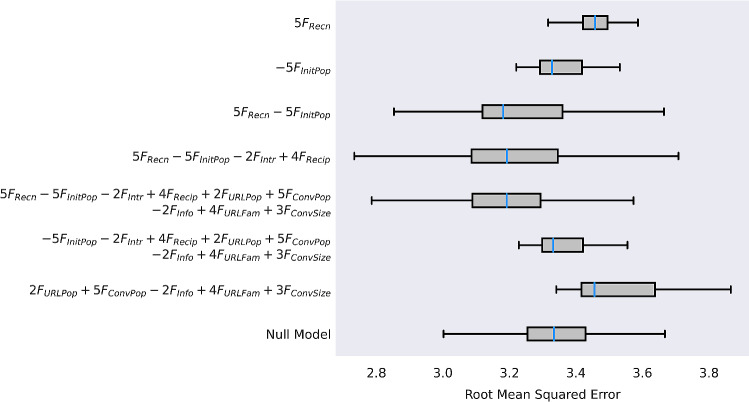
100 runs of MACM-IOM with response prioritization rules inferred through evolutionary model discovery. Simulations of models derived from evolutionary model discovery insights are compared against a null model with random message prioritization. Each model was run 100 times. Only models that include both $F_{Recn}$ and $F_{InitPop}$ have a lower median RMSE than that of the null model.

Finally, we demonstrate the robustness of these results by comparing simulations of models, derived by the above insights, on the holdout users. The derived models were compared against a null model for which all actionable information were shuffled by allocating random priorities to messages by sampling a uniform random distribution. Each model was run for 100 simulations. Comparisons of the RMSE of the resulting simulations are visualized in Figure 6, while Table 2 provides the results of one-tailed Mann–Whitney U tests conducted on the alternative hypothesis that each derived model produced lower median RMSE than that of the null model (significance level = 0.05). Models comprising of only one of the two factors with the highest importance, $u(m) = F_{Recn}$ and $u(m) = F_{InitPop}$, at their optimal presence, did not have lower RMSE than the null model (U = 7736.0, p = 1.0> 0.05 and U = 5566, p = 0.9168 > 0.05, respectively). However, models that included both these factors outperform the null model; $u(m) = 5F_{Recn}-5F_{InitPop}$: $U=3460$, $p=8.4416\times 10^{-5} < 0.05$, $u(m) = 5F_{Recn}-5F_{InitPop}-2F_{Intr}+4F_{Recip}$: $U=3366$, $p=3.2859\times 10^{-5} < 0.05$, and $u(m) = 5F_{Recn}-5F_{InitPop}-2F_{Intr}+4F_{Recip}+2F_{URLPop}+5F_{ConvPop}-2F_{Info}+4F_{URLFam}+3F_{ConvSize}$: $U=2793$, $p=3.4968\times 10^{-8} < 0.05$. When either one or both $F_{Recn}$ and $F_{InitPop}$ are absent from the rule, the derived model does not perform better than the null model, $u(m) = -5F_{InitPop}-2F_{Intr}+4F_{Recip}+2F_{URLPop}+5F_{ConvPop}-2F_{Info}+4F_{URLFam}+3F_{ConvSize}$: $U=5626$, 0.9371 and $u(m) = 2F_{URLPop}+5F_{ConvPop}-2F_{Info}+4F_{URLFam}+3F_{ConvSize}$: $U=8059$, $p=1.0$.

**Table 2:** Comparisons between prioritization rules derived from evolutionary model discovery insights against a null model.

Rule	U	p value
$5F_{Recn}$	7736	1.0
$-5F_{InitPop}$	5566	0.9168
$5F_{Recn}-5F_{InitPop}$	3460	$8.4416\times 10^{-05}$
$5F_{Recn}-5F_{InitPop}-2F_{Intr}+4F_{Recip}$	3366	$3.2859\times 10^{-05}$
$5F_{Recn}-5F_{InitPop}-2F_{Intr}+4F_{Recip}+2F_{URLPop}+5F_{ConvPop}-2F_{Info}+4F_{URLFam}+3F_{ConvSize}$	2793	$3.4968\times 10^{-08}$
$-5F_{InitPop}-2F_{Intr}+4F_{Recip}+2F_{URLPop}+5F_{ConvPop}-2F_{Info}+4F_{URLFam}+3F_{ConvSize}$	5626	0.9371
$2F_{URLPop}+5F_{ConvPop}-2F_{Info}+4F_{URLFam}+3F_{ConvSize}$	8059	1.0

Results for Mann–Whitney U tests for the alternative hypothesis that each derived model had a median RMSE less than that of the null model with random priority allocation (significance level = 0.05). Only models that included both $F_{Recn}$ and $F_{InitPop}$ outperformed the null model.

## Conclusions

We investigated nine factors hypothesized to contribute to message visibility by online social media users under information overload. The Information Overload Model (IOM)^6^ used provides a mechanistic representation of how responsiveness decreases due to increased loss of actionable information under information overload. IOM relates the intensity of actionable information loss to the intensity of overload experienced, via a power-law, by calculating the number of received messages that the agent can process and respond to. Messages are ordered by priority, with lower priority messages being lost first when under overload. While the basic IOM assumes that prioritization is solely dependent on message recency, we relaxed this assumption, and tested alternate utility functions for calculating message priority. Using evolutionary model discovery^21^, we evolved this utility function and examined the importance of the nine hypothesized factors towards the model’s ability to replicate overall responsiveness observed in the data.

Our findings show that, of the factors considered, recency (positive $F_{Recn}$) is in fact the most important factor driving response prioritization. Moreover, messages received more recently are more likely to be responded to by highly active users, despite information overload. This result agrees with findings in the literature that demonstrate the importance of recency in the propagation of news online^20^, and in particular, within Twitter, where information on a user’s timeline may be ordered reverse-chronologically, i.e. starting from the most recent notifications to the oldest^18^. Interestingly, Twitter switched the default timeline ordering from reverse-chronological to an algorithmically determined *most relevant* ordering in 2016, yet later allowed users to switch back to reverse-chronological ordering^48^. An interesting avenue for future work would be to analyze how effective Twitter’s *most relevant* ordering is in providing users with the information that they are most responsive to, in comparison to reverse-chronological ordering.

Next, we find that the popularity of the user initiating a conversation to which a message belongs (negative $F_{InitPop}$) is the second most important factor, which however, has a negative effect on response priority. Also, preference for larger conversation size (positive $F_{ConvSize}$), high conversation popularity (positive $F_{ConvPop}$), and high URL popularity (positive $F_{URLPop}$) were of relatively lower importance. Together, these observations indicate that under information overload, the effects of social learning theory and status theory seem to diminish.

Instead, our results concur that overloaded users are novelty-affinitive^49–52^, exhibit both heterophilous and homophilous behavior^53–56^, and are more responsive within reciprocal relationships^57,58^. The factors encoding these three social theories were found to be of relatively moderate importance, after recency and low popularity of the conversation initiator, leading us to the above conclusion. In particular, messages with content previously discussed by the user were less likely to be responded to (negative $F_{info}$), indicating an affinity for novel information. We found that mechanisms that either prioritized or deprioritized messages from users with common interests (negative or positive $F_{Intr}$) were more likely than those that did not consider common interests at all, indicating that both homophilic behavior, in accordance to social correlation theory, and heterophilic behavior were likely. Messages from senders who were historically more likely to respond back to a responding user (positive $F_{Recip}$) were more likely to gain responses under information overload as well, indicating the importance of reciprocity, and supporting social exchange theory.

Finally, the strong prioritization on message recency and unpopularity of the conversation initiator, by overloaded users, was confirmed by comparing simulations of models including and excluding these two factors, against those of a null model with random prioritization. Models that considered both these factors at optimal presence were able to outperform the null model, while those that did not contain both these factors at optimal presence did not. To summarize, a cognitively plausible mechanism for information overload would prioritize the most recently received messages, posted by less popular users, with a slight tendency to prefer novel interactions that offered high absolute reciprocity, and demonstrated either homophilic or heterophilic tendencies.

## Supplementary information

Supplementary Information 1

## Data Availability

The datasets generated during and analysed during the current study are available in the Open Science Framework repository, https://osf.io/y5swr/?view_only=baaef2d3d11c4aeca5223d124c936bfe.
