# Supplementary material for: Inferring mechanisms of response prioritization on social media under information overload
Source: Sci Rep. 2021 Jan 14;11:1346. doi: 10.1038/s41598-020-79897-5 (PMC7809357; doi:10.1038/s41598-020-79897-5)
Supplement: Supplementary file 1 — Supplementary Information 1 [file 41598_2020_79897_MOESM1_ESM.pdf]

Inferring Mechanisms of Response Prioritization on Social Media under Information Overload

Chathika Gunaratne[1], William Rand[2], and Ivan Garibay[1,*]

[1]Department of Industrial Engineering and Management Systems, Orlando, Florida, 32816, USA
[2]Department of Business Management, Raleigh, North Carolina, 27695, USA
[*]igaribay@ucf.edu

Supplementary Information

Below are histograms displaying the distributions of the data required for the 8 of the 9 hypothesized factors used in the paper over the combined training and simulation periods. F_{Recn} required the the number of simulation time steps a message had remained in an agent's actionable information queue, and therefore did not require any pre-calculations from the data.

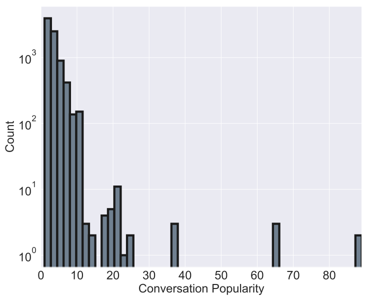

(a) Total number of unique participants per conversation.

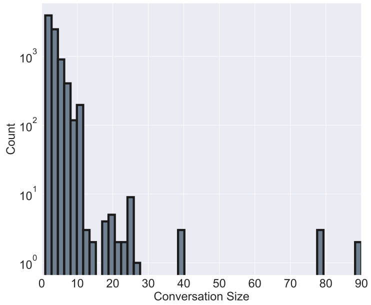

(b) Total number of unique actions per conversation.

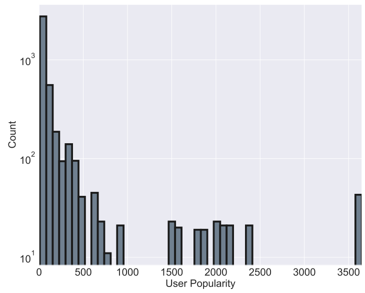

(c) Number of unique user interactions per user.

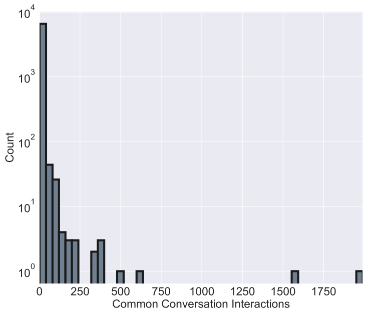

(d) Number of times two users have interacted on the same conversation.

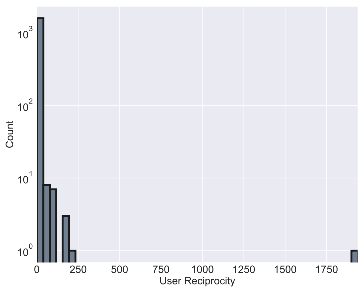

(e) Number of direct responses by user to a particular action by another user.

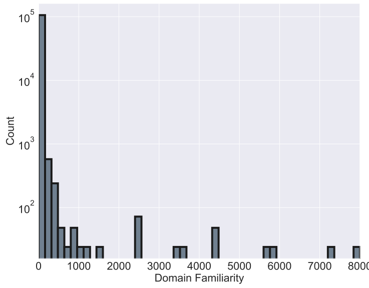

(f) Number of times a particular URL domain was mentioned by a particular user.

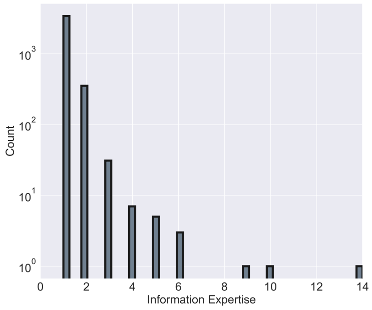

(g) Number of times a particular content keyword was mentioned by a user.

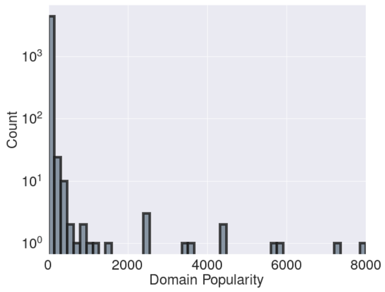

(h) Total number of times a particular URL domain appeared in the data.

Supplementary Figure 1: Histograms of measurements used to calculate subscores for 8 of the 9 hypothesized factors (F_{Recn} didn't require pre-calculated measurements other than the conditional probabilities described in the paper).

Below is an example of a bloated syntax tree produced by the genetic program. Several subtrees have repeated occurrences in the tree, however after simplification this tree reduces to 7 terms: $2(F_{Recn}2F_{Intr}) + (F_{URLFam}F_{Recn}(F_{Intr})^3) + ((F_{URLFam} + F_{URLFam})/F_{URLFam}) + 2(F_{Intr})^2 + 5F_{URLFam} + 28F_{Recn} + 2F_{Intr}$.

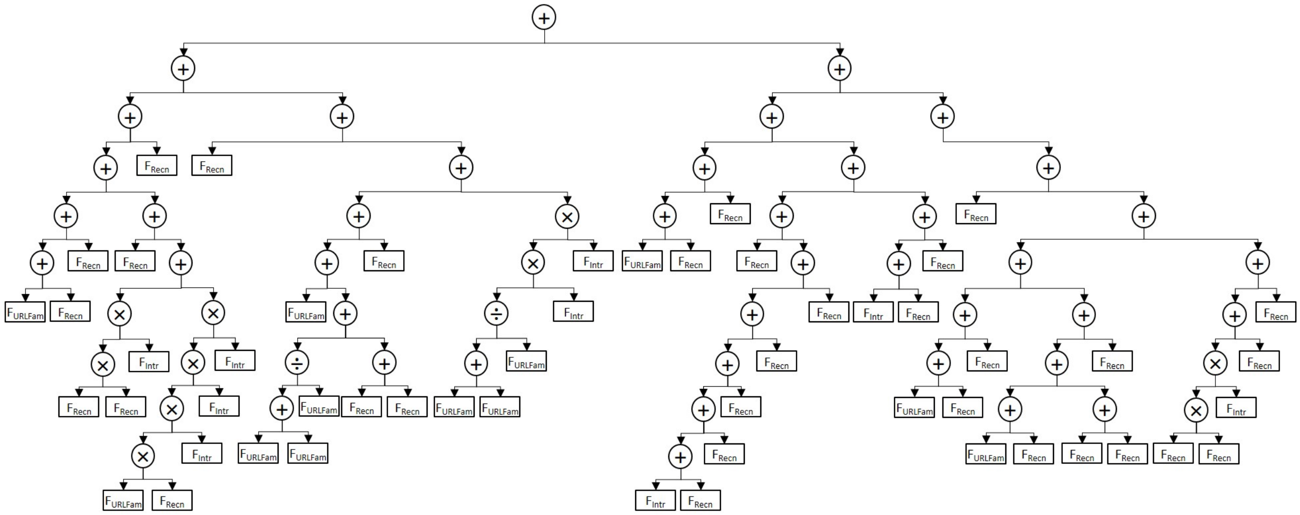

Supplementary Figure 2: Example of a bloated syntax tree produced during by the genetic program. This particular tree reduces into the simplified function: $2(F_{Recn}2F_{Intr}) + (F_{URLFam}F_{Recn}(F_{Intr})^3) + ((F_{URLFam} + F_{URLFam})/F_{URLFam}) + 2(F_{Intr})^2 + 5F_{URLFam} + 28F_{Recn} + 2F_{Intr}$. Having a maximum tree depth of 10 restricted bloating to the intensity displayed.

Below is a plot of the mean RMSE (with ±2 std. dev. in blue) for all 30 genetic program runs over generations.

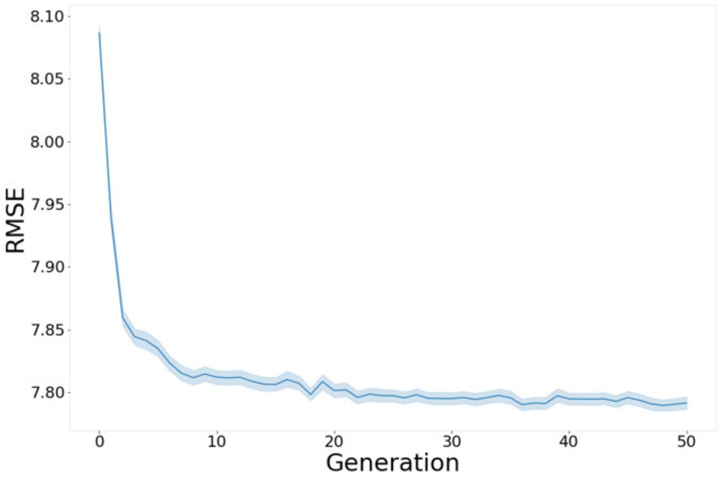

Supplementary Figure 3: Mean Root mean squared error (with ±2 std. dev. in blue), over generations of the genetic program for all 30 runs.
